# Effectiveness of Neurodynamic Interventions in Patients With Stroke: Protocol for a Systematic Review and Meta-analysis

**DOI:** 10.2196/38956

**Published:** 2022-09-07

**Authors:** Abubeker Alebachew Seid, Abdulkerim Hassen Moloro

**Affiliations:** 1 Department of Nursing College of Medicine and Health Sciences Samara University Semera Ethiopia

**Keywords:** stroke, neurodynamic, neurological, physiotherapy, physiotherapist, neural mobilization, review, intervention, search strategy, search strategies, library science, information science, librarian, pain, quality of life, disability, disabilities, physical function

## Abstract

**Background:**

Stroke is the most common and serious neurological condition, which can lead to death, limited functionality, and reduced quality of life. Studies with conflicting results and various methodological limitations have been conducted to assess the effectiveness of neurodynamic interventions for patients with stroke.

**Objective:**

This systematic review and meta-analysis aimed to investigate the pooled effectiveness of different neurodynamic interventions on patients with stroke.

**Methods:**

The PubMed, PEDro, and Google Scholar databases will be searched for studies published with full text in the English language from inception to date. Randomized controlled trials evaluating the effect of different neurodynamic techniques on patients with stroke will be included. The primary outcome measures will include pain, disability/function, and quality of life. Secondary outcome measures will include physical performance measures such as balance, range of motion, muscle strength, and specific diagnostic and neurodynamic test outcomes. The screening, data extraction, and methodological quality assessment will be performed by two independent reviewers. The PEDro scale will be used to systematically appraise the methodological quality. Review Manager V.5.4 software will be used for statistical analysis. Weighted mean difference or standardized mean difference with 95% CIs and *P* values will be used to calculate the treatment effect for each outcome variable.

**Results:**

Search terms and search databases have been identified. The data extraction sheet has also been developed. This study is expected to be completed by the end of 2022.

**Conclusions:**

This study will provide up-to-date evidence on the effectiveness and use of neurodynamic interventions for patients with stroke in clinical practice.

**Trial Registration:**

PROSPERO CRD42022319972; https://www.crd.york.ac.uk/prospero/display_record.php?RecordID=319972

**International Registered Report Identifier (IRRID):**

PRR1-10.2196/38956

## Introduction

According to the World Health Organization, stroke is a condition with no apparent cause other than vascular origin with symptoms lasting for more than 24 hours or leading to death with a rapidly developing clinical sign of focal or global disturbances of cerebral function [1]. Stroke is the most common serious neurological disorder, and in high-income countries, it is the fourth-leading cause of death, long-term disability, and reduced quality of life among adults [1,2]. According to the American Stroke Association, about 87% of the cases are ischemic, and the remaining 13% are hemorrhagic [1]. The most common symptoms include paralysis (in one or both sides), loss of balance, and spasticity, which commonly appear days or weeks after the occurrence of a stroke [3]. Hemiplegia is the primary motor manifestation of stroke, which is weakness of one half of the body contralateral to the site of a cerebral lesion [4].

Several manual therapy techniques were used in the management of patients with stroke including neurodynamic or neural mobilization (NM) techniques. NM is defined as manual techniques or exercise interventions aimed at affecting the neural structures or surrounding tissue (interface) directly or indirectly with the purpose of reducing pain, decreasing neural tension, and improving muscle flexibility and endurance [2,5]. Studies revealed that NM improves the elasticity of nervous and musculoskeletal tissues, increases the intraneural blood flow, improves intraneural fluid dispersion, reduces intraneural edema, reduces thermal and mechanical hyperalgesia, and reverses the increased immune responses following a nerve injury [2,3,5]. NMs restore the mechanical and neurophysiological function of the nerve and can be performed in different ways using active or passive movement, manual mobilization of the nerve or interface, and exercise [6,7].

A systematic review of the literature on the therapeutic efficacy of NM on various musculoskeletal conditions included 10 randomized controlled trials (RCTs) and revealed that there is limited evidence to support the use of NMs [8]. A systematic review of 13 clinical trials focused on carpal tunnel syndrome concluded that the efficacy of NM for carpal tunnel syndrome was unclear [9]. Another review of 20 clinically controlled trials assessed the effect of NM on chronic conditions and concluded that NM is not superior to other interventions [10]. A study conducted to examine the effect of rhythmic upper extremity neurodynamic for 18 patients with hemiplegia caused by stroke found that rhythmic neurodynamic was effective for improving the functions of upper extremities [11]. A blinded randomized clinical trial study on effectiveness of NMs performed in 12 volunteers, aged between 20 and 80 years, with a diagnosis of ischemic or hemorrhagic stroke showed positive effects in relation to flexibility, lower limb muscle strength, gait, and balance [2]. A study on 26 patients with stroke undertaken to compare the efficacy of instrument-assisted soft tissue mobilization and a neural dynamic technique on lower extremity muscle tone, stiffness, and static balance showed a significant improvement in the instrument-assisted soft tissue mobilization group in muscle tone and stiffness but no difference in static balance [3]. A quasi-experimental study to determine the effect of the neurodynamic sliding technique on 20 hemiplegic patients with hamstring tightness showed improved hip flexion assessed by the passive straight leg raise test [12]. A case report study on a combination therapy of botulinum toxin type A and NM for a patient with severe upper limb spasticity and pain after stroke showed an improved joint range of motion and decreased pain, anxiety, and depression [7]. A pretest and posttest experimental study on 20 patients with traumatic spinal cord injury (level C5-C8) and upper limb spasticity in the finger and wrist flexors suggest that neurodynamic mobilization of the median nerve may be effective for upper limb spasticity control and upper limb functional improvements [13].

A systematic review on neurodynamic techniques and mobilization in stroke rehabilitation finally included 12 studies (7 were RCTs, 3 quasi-experimental, 1 case report, and 1 systematic analysis) and concluded that there is limited evidence to support the use of NM techniques [4]. Another review on NM as a therapeutic option in the treatment of stroke included only 6 studies, and the results indicated beneficial effects of NM in the control of muscle tone, range of motion, and functionality of patients affected by stroke [14]. Previous studies on NM interventions on patients with stroke were limited to qualitative analysis, were not up to date, and had unclear or conflicting results [4,5,8,14]. Due to the limited evidence and varying methodological quality, conclusions may change over time [5], and new RCTs are available in the literature. For these reasons, we plan to perform this systematic review and meta-analysis to generate new evidence. The aim of this systematic review and meta-analysis is to systematically assess the types and techniques of different neurodynamic interventions used and their effectiveness on pain, disability, functional status, quality of life, and other variables on patients with stroke.

## Methods

### Overview

**Figure 1:**
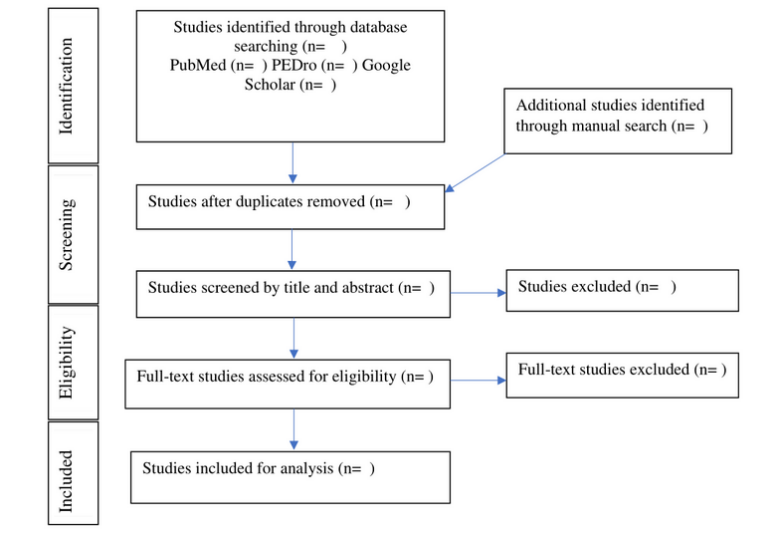
PRISMA (Preferred Reporting Items for Systematic Reviews and Meta-Analyses) flowchart.

This systematic review and meta-analysis protocol is prepared according to the PRISMA-P (Preferred Reporting Items for Systematic Review and Meta-Analysis Protocols) statement [15]. The reporting flowchart is presented in Figure 1. This systematic review is registered on PROSPERO with the registration number CRD42022319972. A preliminary database search and development of a data extraction sheet were undergoing during the submission of this manuscript.

### Search Strategy

To find both published and unpublished studies, a three-step search strategy will be used. An initial limited search of PubMed will be carried out to find relevant Medical Subject Headings (MeSH) and entry terms. A second search using all identified MeSH and entry terms will then be undertaken across the PubMed, PEDro, and Google Scholar databases. Third, the reference lists of all identified studies will be searched for additional studies and articles published from inception to date. The search terms with multiple combinations will be stroke, cerebrovascular disease, hemiplegia, neural, nerve, mobilization, manipulation, physical therapy, physiotherapy, manual therapy, glide, slide, tension, stretching, neurodynamics, and RCTs. Two reviewers will independently screen the titles and abstracts of the studies, and any disagreement between the reviewers will be resolved by consensus or by a third reviewer. The search strategy of the PubMed database is presented in Table 1.

**Table 1:** PubMed search strategy.

Search number	Search detail
1	“stroke”[MeSH Terms]
2	“stroke”[Title/Abstract] OR “apoplexy”[Title/Abstract] OR “cerebrovascular accident”[Title/Abstract]) OR “cerebral stroke”[Title/Abstract] OR “cerebrovascular accident”[Title/Abstract] OR “cerebrovascular accident acute”[Title/Abstract] OR “cerebrovascular apoplexy”[Title/Abstract] OR “cerebrovascular stroke”[Title/Abstract] OR “stroke acute”[Title/Abstract] OR “vascular accident brain”[Title/Abstract]
3	“stroke rehabilitation”[Title/Abstract] OR “mobilization”[Title/Abstract] OR “neural dynamic”[Title/Abstract] OR “neurodynamic”[Title/Abstract] OR “neuro dynamic technique”[Title/Abstract] OR “manual therapy”[Title/Abstract] OR “manipulation”[Title/Abstract] OR “physical therapy”[Title/Abstract] OR “physiotherapy”[Title/Abstract] OR “glide”[Title/Abstract] OR “slide”[Title/Abstract] OR “tension”[Title/Abstract] OR “stretching”[Title/Abstract]
4	#1 OR #2
5	#3 AND #4
6	Limit to “randomized controlled trial” OR “clinical trial” OR “controlled clinical trial”

### Inclusion Criteria

#### Types of Participants

This systematic review will consider studies that include human participants older than 18 years affected by stroke.

#### Types of Interventions

This review considers studies that evaluate neurodynamic interventions performed on patients with stroke. The intervention group (neurodynamic interventions) will be compared to a control group where another or no type of intervention has been performed. NMs are divided into “sliders” and “tensioners.” Sliders will elongate the nerve bed through movement at one joint while moving another joint to relieve tension in the nerve. With tensioners, joints are moved in such a way that the nerve bed is elongated and the tension in the nerves increases [6].

#### Types of Outcomes

This systematic review will consider studies that include the following primary outcome measures: pain (numerical pain rating scale, visual analog scale), disability and function (Disability of the Arm, Shoulder, and Hand Symptom Scale; Neck Disability Index; Roland Morris; Oswestry; Patient Specific Functional Scale), and quality of life (36-Item Short Form Survey, EQ-5D, World Health Organization Quality-of-Life Scale Physical Domain Score). Secondary outcome measures include physical performance measures like balance (Berg Balance Scale), range of motion (inclinometer, goniometer), muscle strength (Oxford grading, dynamometer), sensation (light touch, pinprick, two-point discrimination, thermal pain threshold), specific diagnostic tests (Tinel’s sign, Phalen’s maneuver), and neurodynamic test outcomes (Upper Limb Neurodynamic Test, straight leg raise, slump, prone knee bend, passive neck flexion).

#### Types of Studies

RCTs evaluating the effect of neurodynamic interventions on patients with stroke will be included. Studies that included infants and children, studies with a small sample size, and studies that do not have enough statistical information to be extracted will be excluded. Studies not published in English will be excluded.

### Data Extraction

Data will be extracted independently by two reviewers using a standardized data extraction tool. The data extracted will include specific details about the study methods, populations, interventions, and outcomes of significance to the review question. The data extraction sheet is presented as Multimedia Appendix 1. Authors will check for completeness and any disagreements will be resolved by discussion.

### Methodological Quality Assessment

Papers selected for retrieval will be assessed by two independent reviewers for methodological validity prior to inclusion in the review using the PEDro scale [16]. The possible score on the scale ranges from 0 to 10, with a higher score indicating a higher quality of methods used in the study. A study with a score of 6 or more is considered as evidence level 1 and will be included for data extraction [17]. Any disagreements that arise between the reviewers’ scores will be resolved through discussion or with a third reviewer.

### Statistical Analyses

Review Manager V.5.4 (Cochrane Collaboration) software will be used to analyze the statistical data. We will calculate the treatment effect size as weighted mean difference or standardized mean difference with a 95% CI, and results will be displayed in the form of forest plots. Heterogeneity among included studies will be assessed using the *I*^2^ test. If *I*^2^>0.5 or *P*<.10, the study is considered to have a significant heterogeneity among the included studies, [18] and a random-effect model will be used in this case. Where statistical pooling is not possible, the findings will be presented in a narrative form, including tables and figures to aid in data presentation where appropriate.

### Ethical Considerations

Ethical approval and informed consent are not required, as this study is a literature review that only involves the use of previously published data and does not include any patients.

## Results

The search terms and databases have been identified. After selecting relevant studies and assessing the methodological quality, data extraction and statistical analyses will start. The data synthesis and presentation of the findings will be completed by the end of 2022. The final results will be published in a peer-reviewed journal and will be presented at relevant conferences and events.

## Discussion

This systematic review and meta-analysis will analyze the effects of different neurodynamic interventions on patients with stroke. We will explore their effect on pain, disability/functional status, quality of life, and other variables where data are available. We will also explore the types/techniques and durations of the interventions used. The final results will bring new and up-to-date evidence by investigating the pooled effect of neurodynamic interventions on patients with stroke in the literature. However, this study will have several potential limitations. First, a lack of RCTs with an adequate sample size might be the primary limitation. Second, there may be substantial heterogeneity due to the quality of different studies and due to different methods of neurodynamic interventions (eg, passive vs active approach, sliding vs tensioning techniques, and global vs local tissue mobilization). Finally, some RCTs may be of poor quality, and there may be a potential risk of bias. The final results of this systematic review and meta-analysis will be published in a peer-reviewed journal and presented at relevant conferences and events.

## Appendix

Multimedia Appendix 1 Data extraction sheet (sample).

## Abbreviations

MeSH: Medical Subject Headings
NM: neural mobilization
PRISMA-P: Preferred Reporting Items for Systematic Review and Meta-Analysis Protocols
RCT: randomized controlled trial
