# Supplementary material for: Effectiveness of Neurodynamic Interventions in Patients With Stroke: Protocol for a Systematic Review and Meta-analysis
Source: JMIR Res Protoc. 2022 Sep 7;11(9):e38956. doi: 10.2196/38956 (PMC9494217; doi:10.2196/38956)
Supplement: Multimedia Appendix 1 [file resprot_v11i9e38956_app1.docx]

| **Author (yr.)** | **N** | **Inclusion Criteria’s** | **Exclusion Criteria’s** | **Intervention/s** | **Duration** | **Outcome Measures** | **Intervention group** | | **Control group** | | **Result/conclusion** |
| --- | --- | --- | --- | --- | --- | --- | --- | --- | --- | --- | --- |
|  |  |  |  |  |  |  | **Pre** | **Post** | **Pre** | **Post** |  |
|  |  |  |  |  |  |  |  |  |  |  |  |
|  |  |  |  |  |  |  |  |  |  |  |  |
|  |  |  |  |  |  |  |  |  |  |  |  |
|  | | | | | | | | | | | |

Supplementary file. Data extraction sheet (sample)
